# Acute Patent Ductus Arteriosus Stent Occlusion: A Unique Perspective

**DOI:** 10.1007/s00246-024-03713-4

**Published:** 2024-11-22

**Authors:** Jesse Lee, Shireen Mukadam, Randall Fortuna, Anees Razzouk, Stephen Nageotte

**Affiliations:** 1 Division of Pediatric Cardiology, Loma Linda University, 11234 Anderson St, MC-4434, Loma Linda, CA USA; 2 Division of Pediatric Cardiothoracic Surgery, Loma Linda University, Loma Linda, CA USA

A term female was diagnosed with Tetralogy of Fallot and pulmonary atresia, birth weight of 2.5 kg. CT angiogram showed a long tortuous patent ductus arteriosus (PDA) from the undersurface of the aorta supplying confluent but mildly hypoplastic pulmonary arteries (Video 1). The PDA measured 4.4 mm at the aortic end, 3.1 mm at the pulmonary end, a narrowest constriction of 2 mm in the mid segment, and an estimated total length of 32 mm. Multidisciplinary decision was made to proceed with ductal stenting for pulmonary blood flow supply at 1 month with weight of 2.9 kg.

As the procedure was high risk, we partnered with our cardiothoracic surgeon for a carotid cut-down and ECMO stand-by. Using previously described techniques [1], we were able to negotiate an 0.014 Thruway wire into the right pulmonary artery. We initially attempted to place a long Abbott Xience 3.5 mm × 33 mm stent; however, it would not advance across. At this time, the saturations drifted to 60%; thus, we quickly exchanged for a 3.5 mm × 23 mm stent and deployed it in the distal PDA crossing the pulmonary end. Saturations improved from 60 to 80%. However, there was a lot of PDA uncovered toward the aorta. With some difficulty, a second 3.5 mm × 12 mm length stent was negotiated into place to extend the prior stent proximally with about 4 mm stent overlap. After deployment, the saturations immediately worsened. Angiogram showed acute obstruction in the initial stent (Fig. 1, Video 1). Multiple heparin boluses were given which did not improve patency. Wire position was ultimately lost as attempts were made to intervene on the stent. The patient was emergently cannulated onto VA ECMO and brought to the operating room for emergent shunt placement. The PDA stent was explanted (Fig. 1). Gross evaluation of the stent in the operating room showed protrusion of ductal tissue into the stent without evidence of thrombosis inside the stent. Histologic evaluation was similar. Patient was discharged home 10 days post-surgery.

**Fig. 1:**
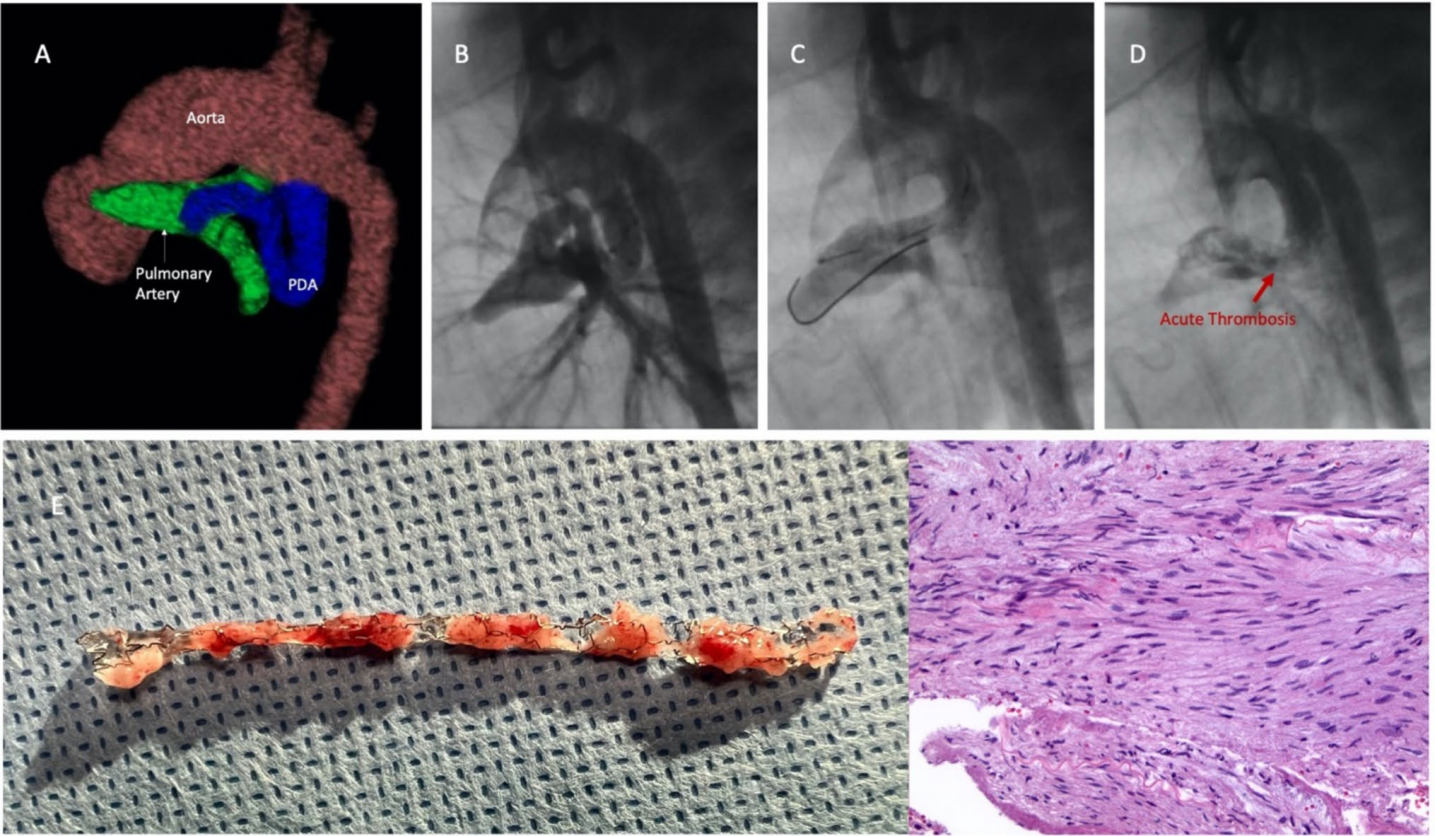
**A** 3D reconstruction from CT angiogram showing tortuous PDA. **B–D** Procedural angiograms from lateral projection showing baseline anatomy, patency after initial stent, and acute occlusion after second stent. **E** Explanted stent with pathologic review showing soft tissue associated with a small portion of vessel wall, blood, and proteinaceous material without evidence of clot organization

Acute ductal stent occlusion has been reported as a rare event [2, 3]. Our pathologic review highlights acute PDA occlusion may result from spasm of friable or injured ductal tissue which would not be amenable to thrombolytics. This is consistent with the literature suggesting re-stent or surgical management is necessary.

## Supplementary Information

Below is the link to the electronic supplementary material.

Supplementary file1 (MP4 9949 KB)

## Data Availability

No datasets were generated or analysed during the current study.

## References

[1] Lee J, Ratnayaka K et al (2019) Stenting the vertical neonatal ductus arteriosus via the percutaneous axillary approach. Congenit Heart Dis 00:1–6 10.1111/chd.12786 31083775

[2] Alwi M (2008) Stenting the ductus arteriosus: case selection, technique and possible complications. Ann Pediatr Cardiol 1(1):38–45 20300236 10.4103/0974-2069.41054 PMC2840730

[3] Bauser-Heaton H, Price K et al (2002) Stenting of the patent ductus arteriosus: a meta-analysis and literature review. J SCAI 1:10039. 10.1016/j.jscai.2022.100392 10.1016/j.jscai.2022.100392 PMC11308046 39132356

